# A person-centred consultation intervention to improve shared decision-making about, and uptake of, osteoporosis medicines (iFraP): a pragmatic, parallel-group, individual randomised controlled trial protocol

**DOI:** 10.3310/nihropenres.13571.3

**Published:** 2025-10-17

**Authors:** Laurna Bullock, Elaine Nicholls, Andrea Cherrington, Stephanie Butler-Walley, Emma M Clark, Jane Fleming, Sarah Leyland, Ida Bentley, Simon Thomas, Cynthia P Iglesias-Urrutia, David Webb, Jo Smith, Sarah Bathers, Sarah Lewis, Angela Clifford, Michele Siciliano, Joanne Protheroe, Sarah Ryan, Janet Lefroy, Nicky Dale, Ashley Hawarden, Sarah Connacher, Robert Horne, Terence W O'Neill, Christian D Mallen, Clare Jinks, Zoe Paskins

**Affiliations:** 1 Centre for Musculoskeletal Health Research, School of Medicine, Keele University, Newcastle under Lyme, England, UK; 2 Keele Clinical Trials Unit, Keele University, Newcastle under Lyme, England, UK; 3 Birmingham Clinical Trials Unit, University of Birmingham, Birmingham, England, UK; 4 Bristol Medical School, Faculty of Health Sciences, University of Bristol, Bristol, England, UK; 5 Cambridge Public Health, University of Cambridge, Cambridge, England, UK; 6 Addenbrooke’s Hospital Fracture Liaison Service, Cambridge University Hospitals NHS Trust, Cambridge, UK; 7 Royal Osteoporosis Society, Bath, England, UK; 8 School of Medicine Research User Group, Keele University, Keele, England, UK; 9 Prescribing Decision Support, Cheshire, UK; 10 Department of Health Sciences, University of York, York, England, UK; 11 Danish Centre for Healthcare Improvements (CHI), Aalborg University, Aalborg, North Denmark Region, Denmark; 12 School of Nursing & Midwifery, Keele University, Keele, England, UK; 13 Haywood Academic Rheumatology Centre, Midlands Partnership University NHS Foundation Trust, Stoke on Trent, UK; 14 Oxford Fracture Prevention & Osteoporosis Service, Oxford University Hospitals NHS Foundations Trust, Oxford, UK; 15 Centre for Behavioural Medicine, UCL School of Pharmacy, University College London, London, England, UK; 16 Centre for Epidemiology Versus Arthritis, The University of Manchester, Manchester, England, UK; 17 NIHR Manchester Biomedical Research Centre, Manchester University NHS Foundation Trust, Manchester, UK

**Keywords:** Shared decision-making, decision aid, osteoporosis, randomised controlled trial, Fracture Liaison Service, iFraP

## Abstract

**Background:**

Good quality shared decision-making (SDM) conversations involve people with, or at risk of osteoporosis and clinicians collaborating to decide, where appropriate, which evidence-based medicines best fit the person’s life, beliefs, and values. We developed the **i**mproving uptake of **Fra**cture **P**revention drug treatments (iFraP) intervention comprising a computerised Decision Support Tool (DST), clinician training package and information resources, for use in UK Fracture Liaison Service consultations. Two primary objectives to determine (1) the effect of the iFraP intervention on patient-reported ease in decision-making about osteoporosis medicines, and (2) cost-effectiveness of iFraP intervention compared to usual NHS care. Secondary objectives are to determine the iFraP intervention effect on patient reported outcome and experience measures, clinical effectiveness (osteoporosis medicine adherence), and to explore intervention acceptability, mechanisms, and processes underlying observed effects, and intervention implementation.

**Methods:**

The iFraP trial is a pragmatic, parallel-group, individual randomised controlled trial in patients referred to a Fracture Liaison Service, with nested mixed methods process evaluation and health economic analysis. Participants aged ≥50 years (n=380) are randomised (1:1 ratio) to one of two arms: (1) iFraP intervention (iFraP-i) or (2) comparator usual NHS care (iFraP-u) and are followed up at 2-weeks and 3-months. The primary outcome is ease of decision-making assessed 2 weeks after the consultation using the Decisional Conflict Scale (DCS). The primary objectives will be addressed by comparing the mean DCS score in each trial arm (using analysis of covariance) for patients given an osteoporosis medicine recommendation, alongside a within-trial cost-effectiveness and value of information (VoI) analysis. Process evaluation data collection includes consultation recordings, semi-structured interviews, and DST analytics.

**Discussion:**

The iFraP trial will answer important questions about the effectiveness of the new ‘iFraP’ osteoporosis DST, coupled with clinician training, on SDM and informed initiation of osteoporosis medicines.

**Trial registration:**

ISRCTN 10606407, 21/11/2022 https://doi.org/10.1186/ISRCTN10606407

## Plain Language Summary

**Background:**

Fragility fractures (or broken bones) can result in significant disability and loss of independence and confidence. Many people who have osteoporosis are not offered, do not start, or do not continue to take medicine to reduce the chance of fractures. Sometimes this is because people with osteoporosis are concerned about the medicine side effects or are unsure about what the benefits are.

To address this, we developed the ‘iFraP intervention’:

1. The iFraP ‘decision support tool’: to support patients and healthcare professionals talk together to make decisions about osteoporosis medicines

2. iFraP training for healthcare professionals to:

a. use the tool in appointments with patients
b. give understandable, clear and consistent information
c. listen to and address patient concerns

The iFraP intervention is being tested in a randomised controlled trial to see if it improves decision-making about osteoporosis medicines. Alongside the trial, this process evaluation will answer questions, such as:

- How do healthcare professionals use the tool and training? What factors influence this?
- What do patients and healthcare professionals think of the tool?
- What do healthcare professionals think of the training?
- What parts of the tool and training work (or do not work)? What factors influence this?
- Does the tool help patients and healthcare professionals to make decisions about osteoporosis medicines, and how?

**Methods:**

We will use the range of approaches:

- Video/audio-recorded appointments
- Interviews with patients and healthcare professionals
- Questionnaires completed by patients
- Information about if, and how, the tool is used in consultations

Findings from each approach will be combined to learn about the iFraP intervention.

**Outputs:**

This process evaluation will help to understand how the iFraP intervention was used and why, how the intervention works (or not), and factors that influence this. Findings will help to learn about how decision support tools can be used in practice.

## Introduction

### Background and rationale

In the UK, three million people are estimated to have osteoporosis ^1^, a condition characterised by low bone density, contributing to over 500,000 fragility fractures (fractures resulting from low or no trauma) per year. Evidence-based treatments, such as bisphosphonates, are recommended by the National Institute for Health and Care Excellence (NICE) for people with osteoporosis and/or a high fracture risk ^2^. Despite being inexpensive, cost-effective, readily available and effective at reducing fracture risk, 25% of people who are offered medication decline it (non-initiation) ^3^. Among those who do start bisphosphonates, few persist for long enough for it to be effective, with persistence estimated at 18% to 75% at one year ^4^. Low levels of osteoporosis medicine initiation and persistence, collectively described as ‘adherence’ ^5^, demonstrates an ‘osteoporosis treatment gap’ ^6^. The ‘treatment gap’ refers to the proportion of people in whom osteoporosis medicine is recommended by clinical guidelines, but do not receive treatment. The treatment gap arises because people at risk are either not identified or offered treatment, or are offered treatment, but decide not to take it. The newly proposed term ‘care gap’ ^7^) instead represents the gap between care offered to people with, or at risk of, osteoporotic fractures, and best practice, person-centred care, respecting that non-adherence might represent an informed choice and, instead of focussing on patient non-adherence, shifts attention to interventions and improvements that target healthcare services and professional’s actions and behaviours.

A good quality shared decision-making conversation involves the patient and clinician working together to make decisions based on evidence (including risks, benefits, and possible options) and discussions about how the medicine ‘fits’ with the patient’s life, preferences, beliefs and values ^8^. Medicine adherence is optimised if a person believes that a medicine is necessary, relevant, safe, and practicable ^9^. This demonstrates how shared decision-making has the potential to support osteoporosis medicine adherence ^10, 11^, by ensuring that the medicine is a good ‘fit’ for the patient ^12^. However, patients often do not feel they have sufficient information to make informed decisions about medicines. A UK population survey of 1188 people with osteoporosis and fragility fractures identified ‘improving access to information from health professionals’, and ‘understanding further the safety and benefit of osteoporosis drug treatments’ as the top two patient priorities for research ^13^. Insufficient or inaccessible patient information that does not address health literacy needs limits patient involvement in the consultation and treatment decisions ^14– 16^. Aligning with this, experts suggest that the osteoporosis care gap, in part, represents poor communication of the benefits and risks of osteoporosis medicines.

NICE’s shared decision-making guidelines recommend that, where available, clinicians should use ‘tools’ to implement shared decision-making – often called decision aids (DAs), conversation aids, or decision support tools (DSTs) - as one part of a ‘toolkit’ alongside other clinician skills ^8^. DSTs are evidence-based tools to help people be involved in decision-making about healthcare options; supporting people to make informed, values-based decisions ^10^. DSTs have been shown to increase patient certainty about decisions (decreased decisional conflict), patient knowledge, and improve the accuracy of risk perception ^10^. Evidence also suggests that shared decision-making is an important mechanism to improve patient uptake of medicines ^11^. There is, therefore, promise that an osteoporosis DST may be beneficial in reducing the osteoporosis care gap. However, existing osteoporosis DSTs have so far not been evidenced to improve adherence and importantly, fail to comprehensively meet international quality standards and patient needs ^17^.

To address the need for an osteoporosis DST that meets international standards and patient needs, we developed the iFraP intervention. Intervention development was guided by the Medical Research Council (MRC) complex intervention development and evaluation framework ^18^ and the three-step implementation of change model ^19^, drawing on theory, empirical evidence, stakeholder engagement and guidance ^20^. The iFraP intervention is a person-centred consultation intervention, consisting of a computerised individualised DST, clinician training in enhanced shared decision-making, risk communication, health literacy and use of the tool, and additional paper and web-based resources for patients and their primary care provider. We hypothesize that the iFraP intervention will improve patient ease in decision-making about osteoporosis medicines (by increasing the extent that the patient was informed and involved in the consultation), facilitate shared decision-making, increase informed medicine initiation and reduce levels of medicine discontinuation. A protocol outlining the studies that underpinned iFraP intervention development is published ^20^, and the results of the development studies are also reported elsewhere ^17, 21– 23^.

The current protocol (version 1.6, 13 December 2023) is described below. Key amendments to the protocol since initial trial registration are highlighted with comments in square brackets.

## Protocol

### Objectives

***Primary objectives***

The primary objectives of this trial are to determine:

1. the effect of the iFraP intervention on patient reported ease in decision-making about osteoporosis medicines.
2. the cost-effectiveness of the iFraP intervention compared to usual Fracture Liaison Services; and the value of acquiring additional information (i.e. value of information (VoI)) on iFraP’s cost-effectiveness.

***Secondary objectives***

The secondary objectives of this trial are to determine:

3. the effect of the iFraP intervention on a range of patient reported outcomes and experience measures including provision of person-centred care, satisfaction with information, and illness and treatment beliefs.
4. the clinical effectiveness of the iFraP intervention on adherence including treatment initiation and discontinuation rates.

Separate protocols will detail the process evaluation and health economic evaluation.

## Methods

### Trial context

This trial takes place in the context of Fracture Liaison Services (FLSs) in England, UK (sometimes referred to as Fracture ‘Prevention’ Services). FLSs enact secondary fracture prevention by systematically identifying adults aged ≥50 years with fragility fractures and conducting bone health assessments. Services are usually nurse or allied health professional-led and address bone health by assessing the patient's risk of falls and future fracture and providing treatment recommendations to the patient and primary care, at one or more consultations, typically 2 months after the fracture.

Service provision varies across FLSs, with services ranging from operating a ‘one-stop shop’ model of care, meaning that, if appropriate, patients have a bone density scan (Dual-energy X-ray absorptiometry [DXA]), nurse assessment, drug treatment recommendation, and blood tests as part of one consultation. Other FLS models may not complete all components for all patients (for example, not all patients receive a DXA scan), or may split these components across multiple appointments, supported by different communication modalities (remote, face-to-face, letter) ^22^.

The trial baseline is taken as the FLS consultation where treatment recommendations are given.

### Trial design

The trial is a pragmatic, parallel-group, individual randomised controlled trial in patients referred to UK FLS, with nested process evaluation and health economic evaluation.

The intervention arms are:

- iFraP intervention (iFraP-i): delivery of the iFraP DST in the FLS consultation by an FLS clinician who has completed the iFraP Consultation Skills Training Course
- Usual FLS NHS care (iFraP-u): current FLS usual care, delivered by an FLS clinician who has *not* completed the iFraP Consultation Skills Training Course

Consenting patient participants referred to FLS will be randomised to one of the intervention arms in the trial using a 1:1 ratio. Data is gathered at baseline (before the FLS consultation) and then at 2 weeks, and 3 months after their FLS consultation, via postal or online questionnaires. REDCap, a secure web-based data collection system, will be used to capture and manage all recruitment and data collection.

We considered randomisation at site, clinician, and patient level ^24^. An individual patient level randomised controlled trial was chosen to minimise (a) disruption of clinician turn over (b) complexity of using multiple sites in a cluster design (c) risk of unbalanced recruitment. Contamination between intervention arms was previously hypothesized as a concern. However, contamination is thought to be minimal as only clinicians delivering iFraP will have access to the computerised DST and receive the iFraP clinician training programme. We will attempt to minimise contamination by excluding patients who have a friend or relative in the study. Evidence of contamination will be explored in the process evaluation (separate protocol).

### Ethics

Ethical approval was obtained from East of Scotland Research Ethics Service (EoSRES) (22/ES/0038). Following initial approval from the Research Ethics Committee (REC), they will continually be informed of all substantial changes to the management of the study. Routine reporting will take place in line with REC requirements.

### Site eligibility and recruitment

To be eligible for this trial, FLSs must:

- decide, recommend, and communicate osteoporosis drug recommendations to patients in a face-to-face and/or remote consultation.
- be situated in England, with a minimum of 2 clinicians.

The Royal Osteoporosis Society (ROS), existing clinical networks and the NIHR Clinical Research Network (CRN) will be used to identify eligible FLSs for the iFraP study. The selection of potential FLS sites will consider capturing variation in the FLS’s service provision (informed by the iFraP national survey of FLSs ^22^) and characteristics of the local population. Identified FLSs (face-to-face or remotely) meet with study team members who fully explain the study and describe the study requirements. Informed consent for FLSs to participate will be provided by the research lead/authorised person in each service, acting as ‘guardian’ for patients in their care, following agreement with the team clarifying willingness to undertake the iFraP intervention. FLS clinician agreement to take part in the trial will be captured as part of the written agreement completed by the research lead/authorised person in each service. FLS consent to participate in the trial is formalised through a Sponsor-site agreement. The number of FLSs approached, declining, or considered not eligible will be recorded.

The research sites receive local management approval, trial specific training and trial administrative procedures prior to the start of recruitment into the trial. FLS clinicians and other team members (e.g. FLS administrators) will also be provided with study-specific training including training on completion of study documentation, using the REDCap database, good clinical practice as applicable to research and the maintenance of the study site file and study records.

FLS clinicians are allocated by the study team to intervention arm or comparator usual FLS NHS care arm, considering seniority and years of experience, to try and achieve balance of skills and experience in both arms of the trial.

### Patient participants

The study population consists of patient participants aged ≥50 years referred to the FLS. The eligibility criteria for patients to take part in the iFraP trial reflects the eligibility criteria for FLS.

***Inclusion criteria***

- Adult patients aged ≥50 years eligible for FLS consultation based on having a previous fragility fracture(s).
- Adult patients able to participate in an FLS appointment (face-to-face or remote consultation) with a participating NHS hospital or associated FLS.

***Exclusion criteria***

- Patients who are unable to give full informed consent or unable to comply with study procedures.
- Patients with a friend or relative in the study (identified through self-report).

***Patient identification, recruitment and consent***

**Figure 1.**
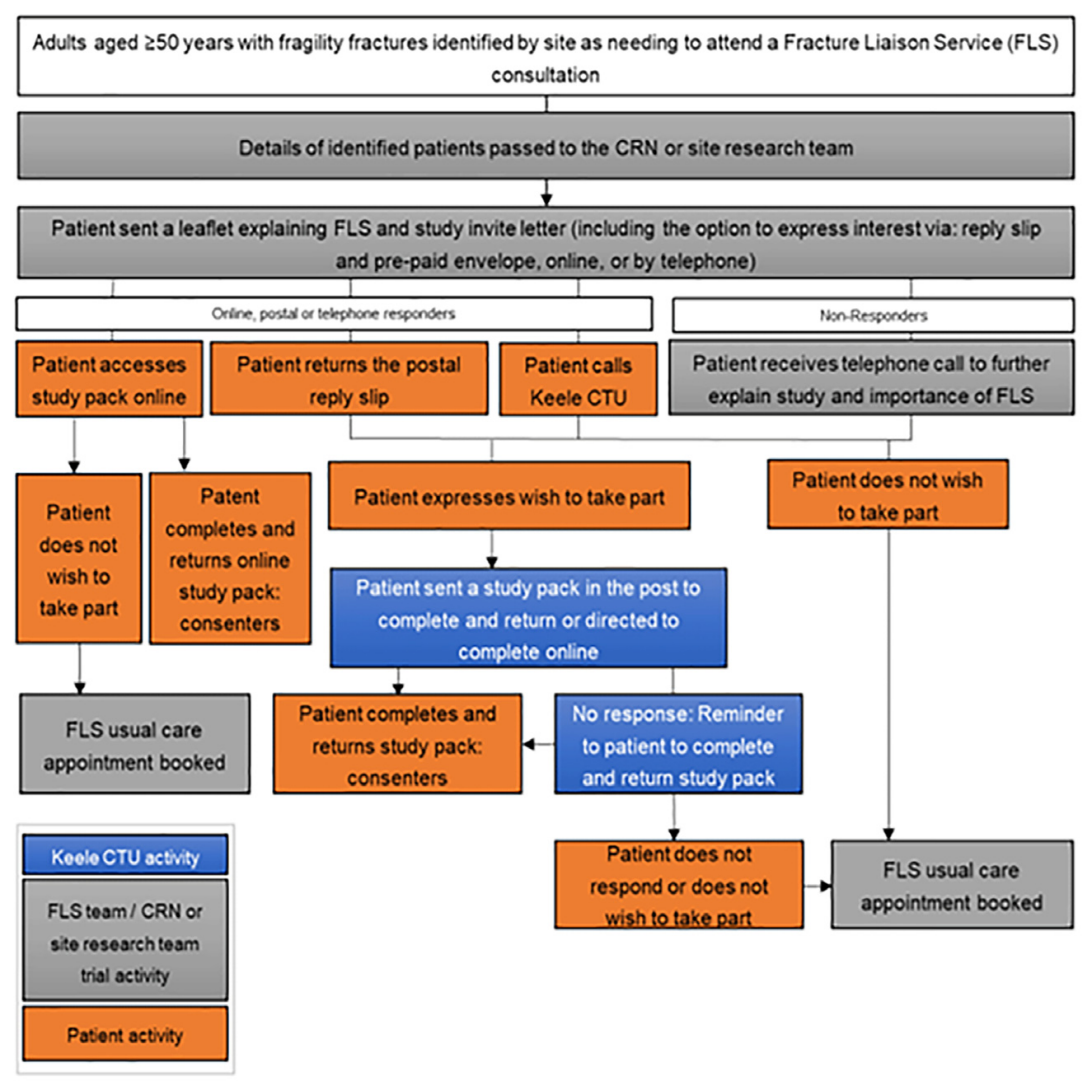
Overview of patient identification.

As part of FLS normal NHS care, adults aged ≥50 years with a recent fragility fracture(s) are systematically identified (primary inclusion criteria). Those that require a face-to-face or remote consultation where medicine may be discussed will be mailed an initial invitation pack, including a flyer explaining that the patient needs a bone health assessment in FLS as part of normal NHS care and a letter introducing the iFraP trial. Age at invite and sex at birth of those mailed will be collected to compare the characteristics of non-responders with responders. See Figure 1 for an overview of recruitment.

Patients interested in hearing more about the study can either access a website (hosted on REDCap), phone, or post back a reply slip to Keele Clinical Trials Unit (CTU) giving consent to contact for more information. Patients that give verbal (via phone) consent to contact, or provide consent to contact using the REDCap or postal forms will be entered onto the Keele study database and allocated a unique participant study ID. Patients who do not respond to the initial mail-out within a defined time window will be telephoned by NHS site staff to explain the purpose of the FLS, explain and gauge interest in the study, and encourage those who do not wish to participate in the study to continue to engage with their normal NHS care FLS appointment.

If the identified patient declines participation or does not respond within an agreed time window, this will be communicated to site allowing an FLS appointment to be booked as per normal NHS care.

Patients that provide consent to contact will be sent the study recruitment pack by post or email, which includes the participant information sheet, consent form, baseline questionnaire and prepaid return envelope (if posted). The study pack includes contact details for the Keele CTU to discuss consent or provide support with data collection, including the opportunity to access translation services.

Patients will be asked to complete and return the study recruitment pack remotely before being randomised and booked an FLS appointment with an appropriate FLS clinician. Remote consent is proportionate with the low-risk nature of the intervention and appropriate for patients that are identified virtually with their consultation by telephone (rendering face-to-face consent inappropriate).

Consent is requested for:

- Taking part in the iFraP trial (read and understood the participant information sheet, voluntary participation, completion of baseline and follow up questionnaires).
- Access to electronic medical records.

Optional consent is also requested to:

- Contact about future related research studies, including the nested process evaluation (e.g. participation in a semi-structured interview, audio/video recording of their FLS consultation) and methodology development.

If the patient completes and returns the study recruitment pack within an agreed time window, they proceed to randomisation. If the patient does not return the pack within a defined period, they will receive a reminder telephone call and/or postcard [Amendment 04, see Table 2]. If the patient does not respond, or declines participation, a non-trial normal NHS FLS appointment will be booked.

At a mid-point in patient recruitment, the Data Monitoring Committee (DMC) will examine the characteristics of the patients recruited to the trial to determine if the sample is representative of the general population. This insight will allow for consideration of approaches to increase diversity [Amendment 08, see Table 2], e.g. whether the NHS site staff could adapt their approach to the introduction telephone calls to focus on underserved groups, who may not be adequately represented.

### Randomisation

***Sequence generation***

The randomisation sequence will be computer-generated (via a computerised random number generator), blocked (random permuted blocks), and stratified by FLS, with an allocation ratio 1:1 and was developed by a database developer with no clinical input to the trial.

***Allocation***

Participants will be randomised by the NHS site team after consent and baseline data collection using the randomisation module in the REDCap database. Once randomised, the authorised staff member at site will be notified by REDCap of the participant’s intervention arm allocation, allowing site to book the appropriate clinic appointment. Emergency telephone backup randomisation will also be available.

***Allocation concealment***

Concealment of the allocation process will be ensured through the remote computer-generation of the randomisation sequence and web-based interface including entry of participant details and necessary consent prior to disclosure of trial arm allocation.

***Blinding***

Participants and clinicians will not be blind to allocation to iFraP intervention (iFraP-i) or comparator usual NHS care (iFraP-u) trial arms. However, any member of the research study team undertaking minimum data collection (MDC) and the statistician will be blind to the trial arm allocation. The qualitative researcher will not be blinded to trial arm allocation.

### Interventions

Patient participants will be allocated to an FLS appointment with an appropriate FLS clinician, based on their randomised allocation. The interventions are to be delivered in a face-to-face or remote FLS consultation where treatment recommendations are given.

***iFraP intervention (iFraP-i)***

The iFraP intervention is a consultation intervention delivered by FLS clinicians to eligible adults aged ≥50 years systematically identified as having a fragility fracture(s), with the aim of facilitating shared decision-making about osteoporosis medicines.

The iFraP intervention consists of three core components:

1. The iFraP DST, used on the computer during the FLS consultation, includes clinician decision-support and a patient-facing DA. It is dynamic, interactive, and tailored to risks and needs of the patient.
  i. First, the clinician enters key patient characteristics into the first part of the DST to receive evidence-based treatment recommendations in line with clinical guidelines. Medicines and Healthcare products Regulatory Agency (MHRA) have advised that the tool is not a notifiable medical device because the tool ‘presents a treatment recommendation informed by national clinical guidelines; that it is a guide only and the clinician ultimately chooses treatment using pre-defined parameters to make a treatment recommendation and is not calculating any new parameters’.
  ii. The second part of the DST is used by the patient and clinician together to navigate discussion about: why bone health is important; the patient’s bone health; and ways to improve bone health, including lifestyle and drug treatment recommendations.
2. iFraP Enhanced Consultation Skills Training Course, completed by FLS clinicians. To decrease the risk of contamination by the sharing of information regarding the intervention between clinicians, we will emphasize to participating clinicians the importance of not sharing information about the intervention with their colleagues during the study. The course includes:
  a. 4-hour interactive eLearning package including expert video presentations and example videos of ‘model’ consultations, with modules introducing the intervention and guidance using the iFraP DST in-practice, risk communication techniques, shared decision-making skills, universal precautions for health literacy and communicating about osteoporosis. At the end of the eLearning course, FLS clinicians are advised to practice using the iFraP DST.
  b. One 3-hour role play session, facilitated by experts in osteoporosis, shared decision-making and consultation communication skills. Each FLS clinician role plays, with their colleagues in-person, as the clinician (using the iFraP DST and implementing eLearning skills) and patient. Facilitators run the role play session remotely, using Microsoft Teams, providing individualised feedback.
3. Information resources (paper and online) for the patient and General Practitioner (GP) to refer to after the FLS consultation. This includes a dentist card that the patient can show to their dentist to support conversations about osteoporosis medicine (including the patient’s medicine start date, expected treatment duration, and who the dentist should contact if they have questions) and an individualised A4 PDF output (described as the ‘personal Bone Health Record’) from the iFraP DST. The Bone Health Record includes answers to questions the patient and clinician complete together, and a URL linking the patient to more information online, including a video of the iFraP DST being demonstrated and explained.

Participants randomised to iFraP-i will be scheduled their FLS appointment with an FLS clinician that has completed the iFraP Consultation Skills Training Course. iFraP-i is delivered in one consultation which may be conducted face-to-face or remotely by video or telephone, depending on a variety of local factors including service commissioning, staffing, and the impact of COVID-19, usually lasting approximately 30 minutes. During the appointment, the FLS clinician will use the iFraP DST and provide the patient with iFraP information resources, such as an individualised summary of the appointment (the ‘personal Bone Health Record’), printed from the iFraP DST in-person (if consulting face-to-face), or sent by post (if consulting remotely). After the consultation, the Bone Health Record will also be shared with the patient’s GP.

***Comparator usual FLS NHS care (iFraP-u)***

Participants randomised to this comparator arm will be scheduled their face-to-face or remote FLS appointment with an FLS clinician delivering usual FLS NHS care. At present, usual FLS care does not use Decision Support Tools to support patient-clinician discussion, nor do FLS clinicians have access to the iFraP Consultation Skills Training Course or information resources that would be provided as part of the iFraP intervention. FLS clinicians delivering iFraP-u will be offered the opportunity to partake in the intervention training at the end of the trial.

***Crossover and post-trial care***

Participants cannot crossover from one arm of the trial to the other. Any protocol deviations that occur during the trial will be recorded and their impact assessed for future trial conduct and the final data analysis plan.

Participants’ clinical care after the FLS appointment returns to normal FLS NHS care.

### Clinical champion

One FLS clinician at each site will be a ‘Clinical Champion’ [Amendment 02, see Table 2] to facilitate the implementation of the iFraP intervention. The Clinical Champion’s role will be to promote the iFraP training and use of the iFraP tool locally, mentor iFraP-i clinicians, and act as a link between the FLS team and the Keele University research team. An introduction session with the Keele University research team will outline the Clinical Champion role and responsibilities, with subsequent monthly meetings attended by all Clinical Champions and Keele University research team to share learning, achievements and overcome challenges. To support this role, the Clinical Champion will take part in the iFraP Consultation Skills Training Course and therefore will not deliver FLS NHS usual care (iFraP-u) appointments to avoid contamination. To support implementation of the iFraP training, email reminders and newsletters will also be sent to participating intervention clinicians.

### Data collection

All participants will be asked to complete 3 paper or online (via REDCap) questionnaires, depending on their specified preference. Questionnaires collect data at baseline (before randomisation), 2 weeks and 3 months after FLS consultation.

Case Report Forms (CRFs) will all be completed electronically using REDCap.

***Baseline data collection***

Baseline data collection will be collected before randomisation, as part of the recruitment pack. Data includes demographic characteristics (date of birth, sex at birth), ethnicity, socioeconomic status (Index of Multiple Deprivation), employment status, marital status, fracture site and risk factors, health literacy ^25^, barriers to communication and first language, digital access, experience of osteoporosis medication. Additional measures included are outlined in Table 1.

**Table 1.** Patient participant questionnaire content.

Trial assessments	Baseline	2-week	3-month
Demographics (date of birth, sex at birth)	✓	✓ *	✓ *
Employment status	✓		
Marital status	✓		
Fracture occurrence including site and date	✓		✓
Fracture risk factors:			
• self-reported height	✓		
• rheumatoid arthritis	✓		
• family history	✓		
• secondary causes see FRAX https://www.sheffield.ac.uk/FRAX/)	✓		
Ethnicity	✓		
Health literacy ^25^	✓		
Barriers to communication (hearing, vision) and first language	✓		
Digital access	✓		
Experience of osteoporosis medicine	✓		
Socioeconomic status (IMD)	✓		
Beliefs about medicines (BMQ-general) ^27^	✓		
**Primary outcome**			
Decisional conflict ^26^		✓	
**Secondary outcome measures for all**			
Modified Patient-Professional Interaction Questionnaire (PPIQ) ^28^ (see extended data)		✓	
Satisfaction with verbal information ^29^ and experience		✓	
Recall of, and satisfaction with written information ^29^			✓
Recall of consultation – including key elements included in the training, being shown the computer, receiving diagnosis, receiving drug recommendation		✓	
Modified Brief Illness Perceptions Questionnaire ^30^ (see extended data)	✓	✓	✓
Self-reported change in physical activity			✓
Worry about further falls and fractures ^31^	✓	✓	
Self-perceived fracture risk ^32^	✓	✓	
Self-reported weight	✓		✓
Alcohol	✓		✓
Smoking	✓		✓
**Secondary outcomes: Recommended medication only**			
Beliefs about medicines (BMQ-specific) ^27^			✓
Satisfaction with medicines information (SIMS) ^33^		✓	
Osteoporosis specific values		✓	
Self-reported medicine initiation or intention to initiate		✓	✓
Self-reported adherence ^34^ and, persistence or discontinuation with medicine			✓
Medicine self-reported side effects	✓		✓
**Health Economic Outcomes**			
Health status – EQ-5D-5L 35	✓	✓	✓
Health care utilisation	✓		✓

*date of birth and sex at birth collected to verify identity at 2 weeks and 3 months

**Table 2.** Protocol amendments since trial registration.

Amendment Number	Protocol Section	Original protocol	Amended details	Date of Amendment REC/HRA approval	Rationale for amendment
AM02 NSA02	iFraP intervention training Patient identification *Nested studies*	Omission of iFraP clinical champion role Patient variables described as ‘sex’ and ‘age’ *[Interviews with primary care clinicians could* *be completed in-person or remotely (by* *telephone or Microsoft Teams)]*	Additional detail about Clinical Champion role included Minor changes to variable names: ‘sex at birth’ and ‘age at invite’, update Trial Manager and timeline *[Primary care clinician interview* *method to remove face-to-face option]*	Dec 2022	To describe FLS Clinical Champion role and clarify patient variables *[The HRA queried the number of GP sites involved* *in the study – this was not possible to predict* *given that the location/site of each primary care* *participant would not be known in-advance.* *Given the geographical spread of potential* *primary care practitioners, interviews were likely* *to be remote.]*
AM04 NSA04	Medical record review *[Nested studies]*	Discontinuation of osteoporosis medicines in medical record review determined by last date of prescription ≥ 6 weeks prior to MRR = yes *[A member of the FLS clinical team to upload* *consultation recording to Keele CTU]* *[Informed consent for face-to-face interviews* *to be gained in-person prior to interview]*	Additional method of identifying discontinuation in medical records: recorded discontinuation in the patient medical record = yes *[An NHS site staff member to upload* *consultation recordings to Keele CTU]* *[Informed consent for face-to-face* *interviews can be obtained in-person* *or remotely prior to interview]*	April 2023	A review of medical records revealed that discontinuation can be explicitly recorded (in addition to only inferring from date prescription filled) *[Greater flexibility required so that NHS research* *teams can support the uploading of consultation* *recordings, where needed.]* *[iFraP-i clinicians can consent to interview and* *consultation recording in one process. Remote* *consent to additional data collection enables* *consultation recordings to begin even if the* *interview has yet to be scheduled.]*
AM07 NSA 07	Patient identification	If a patient provides consent to be contacted, Keele CTU send a study recruitment pack containing the consent form and baseline questionnaire, using their preferred method. If the patient does not return the consent form and/or baseline questionnaire within an agreed time window an FLS appointment will be booked, as per normal NHS care.	A courtesy phone call to be made with patients who consent to be contacted but who do not return the consent form and baseline questionnaire within a defined time window. If the patient cannot be contacted by telephone, they will be sent a reminder postcard.	August 2023	When reviewing recruitment rates, there was evidence that a portion of patients were consenting to contact but not returning the consent form and/or baseline questionnaire. This additional telephone call and/or postcard aimed to improve consent rates.
AM08 NSA 08	Patient identification Follow up assessments	Patient identification: At a mid-point in patient recruitment, the Data Monitoring Committee (DMC) will examine the characteristics of the patients recruited to the trial to determine if the sample is representative of the general population. Dependant on results, NHS sites will be asked to adapt their approach to focus recruitment to underserved groups. Follow up assessments: Non-responders to the 2-week and/or 3 month questionnaires will receive their first reminder after approximately 10 days, as per their contact preference.	Patient identification: Flexibility to consider alternative approaches to recruit underserved groups. Follow up assessments: Non- responders to 2-week and/or 3 month questionnaires, whose preference is for online follow ups, will be sent an additional postal reminder if the follow up questionnaire is not returned.	November 2023	The sample at interim review was 100% self- reported White British. In efforts to address this lack of diversity, an additional FLS site joined the trial (from 3 sites to 4 sites). The FLS was chosen because it served a larger proportion of underserved groups, comparatively to other enrolled FLSs. Wording was changed to allow alternative approaches other than asking NHS sites to adapt their approach to focus recruitment to underserved groups, because it was identified that this may be difficult if ethnicity was not recorded well in medical records. Recruitment and follow up rates were identified as lower than expected. Additional postal reminders for those completing online also aimed to improve follow up rates.
AM09 SA09	Sample size	The original target sample size was 328 patients to achieve 200 patients completing the primary outcome at 2 weeks. Patients will be recruited during an 8-month recruitment period. End of study date = September 2024	The amended target sample size is 380 to achieve 200 patients completing the primary outcome at 2 weeks. Patients will be recruited during an 11-month recruitment period. End of study date = May 2025.	February 2024	The original sample target assumed that approximately 32% of patients would not receive a medicine recommendation (hence for whom the primary outcome is not relevant) and for 10% loss to follow-up in the primary outcome at 2-weeks. Resulting in 200 patients completing the primary outcome. When reviewing recruitment and follow up rates with the DMC and TSC, it was identified that 27% of patients did not receive a medicine recommendation, 20% loss to follow-up in the primary outcome at 2-weeks and for 10% loss due to ‘Do Not Attends’ (DNAs). A target sample of 380 was required to meet 200 responses in the primary outcome.

Amendments related to the process evaluation are provided in *italics* and square brackets. Amendments 1, 3, 5 and 6 are not included here as they did not have implications for the trial protocol

***Primary outcome measure***

Patient reported ease in decision-making about osteoporosis medication, collected at 2 weeks, measured using the Decisional Conflict Scale (DCS) ^26^. The DSC will be completed by those recommended an osteoporosis medicine, using 16 items each measured using 5 Likert response categories from ‘strongly disagree’ to ‘strongly agree’.

***Secondary outcome measures***

The secondary outcome measures collected at 2-weeks and/or 3 months following FLS consultation, completed by all participants, include:

- patient reported perception of patient centred care at 2 weeks using Patient-Professional Interaction Questionnaire (PPIQ) ^28^, modified to i) improve readability, ii) ensure relevance for non-face-to-face consultations, iii) remove binary (he/she) gender pronouns (see extended data)
- worry about further falls and fractures at 2 weeks ^31^
- satisfaction with the consultation and general information using the Satisfaction with Cancer Information Profile scale (SCIP) ^29^ (modified to refer to ‘osteoporosis’ and ‘bone health’ rather than cancer). Satisfaction with verbal information will be assessed at 2 weeks and satisfaction with written information assessed at 3 months.
- fracture risk perceptions at 2 weeks ^32^
- illness perceptions at 2 weeks and 3 months, modified from the Brief Illness Perception Questionnaire ^30^ with public contributors to focus on ‘broken bones’ and ‘bone health’ rather than ‘illness’ (see extended data).
- Self-reported smoking, alcohol, weight and change in physical activity at 3 months.
- Health related quality of life (EQ-5D-5L ^35^) at 2 weeks and 3 months
- recollection of FLS consultation content at 2 weeks, including key elements of the training in the FLS consultation, whether they received a diagnosis of osteoporosis, or a drug treatment recommendation.
- healthcare resource use at 3 months, including healthcare professional contacts, medicines and supplements use.

Additional secondary outcome measures will be self-reported at 2 weeks and/or 3 months by participants recommended osteoporosis medication during their FLS consultation. These include:

- specific osteoporosis values at 2 weeks, including 5 bespoke questions about the relative perceived importance of osteoporosis medicine benefits (e.g. “how important are these treatment benefits to you e.g. maintaining independence”) and possible side effects and adverse events (e.g. “How likely is it, that you would be put off taking this treatment, because of concerns about e.g. common side-effects with medicines such as indigestion and reflux”) using 5-response categories, from “not at all” to “extremely"
- satisfaction with Information about Medicine (SIMS) ^33^ at 2 weeks
- beliefs about medicines (BMQ-specific ^27^) at 3 months
- self-reported medicine initiation or intention to initiate at 2 weeks and 3 months and persistence or discontinuation at 3 months.
- self-reported adherence (Medication Adherence Report Scale ^34^) at 3 months
- self-reported medicine side effects at 3 months

Table 1 summarises the content of the participant questionnaires at baseline, 2-weeks, and 3-months.

***Follow up assessments***

Non-responders to the baseline questionnaire will not be enrolled into the trial and will be allocated a non-trial normal NHS care FLS consultation.

Non-responders to the 2-week and/or 3-month questionnaires will receive a reminder by post and/or email, after approximately 10 days [Amendment 08, see Table 2]. Non-responders to the reminder will be telephoned (by a blinded trial administrator) after approximately 10 days for MDC. A brief questionnaire for MDC will be sent by post or email to those who cannot be contacted after 3 telephone attempts, as per participant’s preference. The MDC questionnaires aim to collect the primary outcome (if appropriate), health-related quality of life (EQ-5D-5L) and self-reported medicine use, along with date of birth and sex at birth to ensure the data are provided by the intended participant.

The flow of events as participants proceed through the trial is outlined in ( Figure 1) and the timing of key events outlined in Table 1.

***Medical record review***

Hospital Medical Record Review (MRR) will be completed 3 months after the FLS appointment. Prescription data, including the number initiated (prescribed osteoporosis drug treatment since FLS date: yes/no) and number discontinued (determined by last date of prescription ≥6 weeks prior to MRR or recorded discontinuation in the patient medical record = yes) will be captured [Amendment 04, Table 2]. Medical record review will also capture information about appointments/visits related to bone health.

### Adverse events

The clinical management recommendations given to participating FLS clinicians, as part of the iFraP DST, are evidence-based best practice, following national guidelines and in line with normal NHS care. Therefore, adverse events in this trial are expected to be minimal. Side effects of osteoporosis medications will be captured as an outcome but not reported as Adverse Events in this trial.

All serious adverse events (SAEs) either confirmed or suspected will be communicated to Keele CTU within 24 hours. An authorised medic would then conduct a causality assessment. Suspected or confirmed SAEs are to be reviewed by the DMC and reported to the Trial Steering Committee (TSC).

### Sample size

To address the objectives of the trial, 380 participants (190 in each intervention arm) need to be recruited to detect a between group effect size of at least 0.4 in the primary outcome at 2-week follow-up, with 2-tailed 5% significance and 80% power [Amendment 09, see Table 2 and progress of the trial]. This sample size target of 380 has been calculated based on estimations that 200 participants will complete the primary outcome at 2 weeks (100 per intervention arm). It is expected that approximately 27% of patients will not receive a medicine recommendation (hence for whom the primary outcome is not relevant), 20% loss to follow-up in the primary outcome at 2-weeks, and 10% loss to follow-up because of ‘Do Not Attends’ (DNAs).

With an estimated standard deviation of 15, this translates to minimum clinically important difference (MCID) of 6 points on the DCS (scale range 0 – 100) – a difference considered to be meaningful and one that produces an effect size in the range of meaningful effect sizes recommended by the authors of the DSC ^26^.

### Analysis methods

Separate a priori analysis plans will be written to describe all pre-planned trial analysis, including clinical and cost-effectiveness of the iFraP intervention alongside the process evaluation. The analysis plans will be agreed and signed off by the TSC and DMC prior to lock down of the final dataset and will be the definitive version. Consequently, only a brief outline of the planned analysis is given here.

***Statistical analysis***

A CONSORT flow diagram will describe the flow of participants through the study and will include reasons for study withdrawal if given. Descriptive statistics will be used to describe the key baseline characteristics of participants at each stage of recruitment and follow-up, and by intervention arm, to assess if there is any evidence of selection bias and to evaluate the success of the randomisation procedure.

Analysis of covariance (ANCOVA) will be used to analyse the primary outcome (the total DCS score) at the 2-week primary endpoint, by comparing the mean outcome in each intervention arm, after adjustment for any pre-specified baseline covariates. A sensitivity analysis of the primary outcome model will be conducted to explore whether study conclusions change when outcome variation between FLS clinicians is accounted for in the model. This will be achieved by adapting the ANCOVA model into a mixed model framework and incorporating a random effect term to represent the clinician who treated the patient. The magnitude of the treatment effect from this model will then be compared to that from the primary analysis in the study. We will consider using multiple imputation to impute the patient-level missing data if the missing data rate is greater than 5% for at least one patient-level outcome or predictor of interest. If multiple imputation is used, this will be regarded as the primary analysis over a complete-case analysis.

Treatment effects for secondary outcomes measured at a single follow-up time-point will be explored using similar methods to the primary outcome analysis, but with ANCOVA, logistic and ordinal regression used as appropriate for continuous, binary, and ordinal outcomes. For outcome measures collected at more than one time-point, linear mixed models will be used to model change in the outcome over time. Results will be presented either as mean or percentage differences/odds ratios alongside their associated 95% confidence intervals.

Descriptive statistics (numbers and percentages) will be used to describe patients experience of their FLS appointment and exploratory analysis conducted to explore whether patients’ perception of their fracture risk changes following the intervention, and whether their post intervention perception of risk is more in line with their predicted fracture risk (as calculated by their Fracture Risk Assessment (FRAX®) score. In addition, we will also explore whether patients’ level of worry about falls and fractures changes following their FLS consultation and whether such changes are similar in both trial arms.

***Economic evaluation***

The economic evaluation will comprise a within-trial cost-effectiveness and value of information (VoI) analysis to determine whether the iFraP intervention is cost-effective compared with usual care. The health economic evaluation protocol will be published separately.

***Process evaluation***

A mixed methods process evaluation, in line with the MRC guidance ^36^, will explore perceived acceptability of iFraP amongst patients and clinicians, what components of iFraP were delivered and how (fidelity), how much of iFraP was delivered (dose), whether iFraP results in a change in outcomes (e.g. shared decision-making, medicine initiation rates) and how, context affects implementation of iFraP and outcomes. Various methods, including semi-structured interviews, CRFs, consultation recordings, and DST analytics will capture data for the process evaluation. The process evaluation protocol will be published separately.

### Patient and Public Involvement

A group of public contributors with osteoporosis and their carers was convened to support the development of the iFraP research programme and the NIHR funding application. The group met prior to funding, helping to define the research questions, and influencing research design. Members of this group have subsequently been invited to form a Patient Advisory Group (PAG) to support delivery of the iFraP research programme. The PAG met throughout the iFraP intervention development work, as outlined elsewhere ^20^. More recently, the PAG have also supported design of the trial, by discussing appropriate outcome measures, piloting the 2-week follow up questionnaire, and commenting on other patient-facing documents. The PAG continue to work with the research team throughout the trial and one member of the PAG has joined the trial study team, regularly attending monthly Trial Management Group (TMG) meetings. Two independent public contributors attend Trial Steering Committee meetings, to ensure public involvement oversight and monitoring of the trial.

### Community of Practice

Communities of Practice (CoPs) bring together expertise with a common concern or interest, with the aim of improving and learning to do better through regular group interaction ^37^. iFraP CoP members include FLS clinicians, GPs, osteoporosis specialists, patients with experience of using osteoporosis medicines (supported by a public involvement worker), representatives from the ROS and Health Literacy UK and a behaviour change expert. The iFraP CoP met regularly throughout the research programme and will continue to meet during the iFraP trial (e.g. to discuss knowledge mobilisation and dissemination).

### Trial organisation and monitoring

The iFraP TMG have overall responsibility for the clinical set-up, promotion, ongoing management and monitoring of the study, and for analysis and interpretation of results. The TMG meet on a regular basis throughout the study. The TMG will monitor protocol compliance of recruitment, treatment, and follow-up procedures during conduct of this study, and this will be discussed at monthly TMG meetings.

The independent TSC provides overall supervision of the research programme according to agreed timelines. The TSC met prior to ethics application to agree the trial protocol, and at agreed time intervals over the course of the trial. An independent DMC approved the protocol and will monitor trial data. Detailed reports focusing on recruitment, retention, protocol compliance, and adverse events are prepared by Keele CTU.

All data collection, database design, data input and cleaning, as well as trial oversight procedures, are in line with the standard operating procedures of Keele CTU and the conditions of the grant. Data will be centrally monitored for quality and completeness by Keele CTU.

### Data confidentiality and archiving

All information collected during the trial will be kept strictly confidential. Information will be held securely on paper and managed electronically using REDCap through Keele CTU. Keele CTU achieve the legal obligations set by the Data Protection Act (2018) and the General Data Protection Regulation (GDPR). If a participant withdraws consent from trial intervention and/or further collection of data, their data will remain on file and is included in the final study analysis. At the end of the trial, data will be securely archived in line with the Sponsor’s procedures for a minimum of 10 years. Data held by Keele CTU will be archived in the designated Keele CTU archive facility and site data and documents will be archived at the participating sites. Following authorisation from the Sponsor, arrangements for confidential destruction will then be made.

Any subsequent requests for access to the data from anyone outside of Keele CTU (e.g. collaboration, joint publication, data sharing requests from publishers) will follow Keele University’s standard operating procedure.

### Progress of the trial

Recruitment commenced in May 2023. A review of recruitment and follow up rates with the DMC and TSC in July 2023 and with the TSC in October 2023 identified slower recruitment and lower follow up rates than anticipated. The TSC recommended that an additional FLS site is recruited to participate in the trial. This resulted in three key amendments to the number of sites, target sample size and recruitment duration and rate:

1. On 14th July 2023, the REC approved an amendment to include one additional FLS research site, increasing the number of participating FLS sites from three to four.
2. The original protocol inflated the sample size of 200 to a target of 328 patients. This target assumed that approximately 32% of patients would not receive a medicine recommendation (hence for whom the primary outcome is not relevant) and accounted for 10% loss to follow-up at 2-weeks. When reviewing these assumptions with the DMC and TSC, approximately 27% of patients did not receive a medicine recommendation, 20% were loss to follow-up at 2-weeks and a further 10% losses were observed due to ‘Do Not Attends’ (DNAs). Therefore, to achieve 200 responses to the primary outcome at two weeks, the sample size target was inflated to 380.
3. During the first 6 months of trial recruitment an average of 27 patients per month were recruited across three sites. Following an addition of an extra site, an amended total recruitment period of 11 months is required to meet the revised sample size (n=380). The monthly target for recruitment was therefore adjusted from 63 to 44.

Protocol amendments are described in detail, along with rationale for change, in Table 2.

### Dissemination

The iFraP trial results will be submitted to peer-review journals for consideration. The research team will also submit abstracts to national and international conferences. Established clinical and third-sector networks will facilitate dissemination to clinical audiences. Dissemination plans will be discussed with public contributors to ensure that results are understandable and reach lay audiences. Patient participants will be informed about the results of the trial at the end of the study.

The iFraP intervention resources will be made freely available on the Evidenced Resources for Osteoporosis (ERO) website — a partnership between the Royal Osteoporosis Society and Keele’s Impact Accelerator Unit to curate, disseminate and increase the impact of osteoporosis evidence-based resources.

## Conclusions/discussion

This paper describes the design of a pragmatic randomised controlled trial which investigates the experience of care and effectiveness of the iFraP intervention compared with FLS usual NHS practice.

A Cochrane review of DAs identified only 10 studies (of 105 identified, 10%) examining the effects of the DA on patient treatment uptake ^10^. The pooled analysis of five studies identified that, when discussing preventative medicine decisions (e.g. to manage type II diabetes), participants exposed to the DA showed a statistically significant increase in medication initiation compared to usual care. The iFraP trial will build on this limited evidence base to understand the clinical effectiveness of a shared decision-making intervention on medicine initiation in osteoporosis. We do acknowledge, however, that the 3-month follow-up period of this trial may limit our understanding about long-term persistence or discontinuation of osteoporosis medicines.

DAs and DSTs have often been described as ‘requiring minimal training for use’. However, to promote successful implementation of shared decision-making in routine clinical practice, it is important those delivering the DST ‘buy-in’ to shared decision-making and are provided with adequate shared decision-making skills training ^38^. iFraP intervention development work identified the need for, and content of, a theoretically-informed clinician training package to be delivered alongside the DST, to support implementation of shared decision-making. The iFraP trial will expand the current evidence by examining the effectiveness of a shared decision-making intervention package (including training), rather than DST in isolation.

It is important that the results of this trial are representative of the people attending FLSs. Data collection materials and methods were carefully considered, with public contributors, in efforts to recruit a diverse trial sample. The public contributors recommended that the trial invite letter included a translated sentence in the five most commonly used languages and additional costs acquired for translation services. In a recent survey of patients attending rheumatology services (mean age 64.5), 20% of respondents reported not having access to an internet-enabled device and 18% reported limited health literacy ^39^. This demonstrated the need for paper surveys (in addition to online surveys) to overcome barriers to participation, despite the additional work required.

## List of abbreviations

**Table:** 

BMQ	Beliefs about Medicines Questionnaire
CI	Chief Investigator
CoP	Community of Practice
CRF	Case Report Form
CTU	Clinical Trials Unit
DA	Decision Aid
DCS	Decisional Conflict Scale
DMC	Data Monitoring Committee
DNA	Do/Did Not Attend
DST	Decision Support Tool
ERO	Evidenced Resources for Osteoporosis
FPS	Fracture Prevention Service
FLS	Fracture Liaison Service
FRAX®	Fracture Risk Assessment Tool
GDPR	General Data Protection Regulation
GP	General Practitioner
ID	Identification
iFraP	**i**mproving uptake of **Fra**cture **P**revention drug treatments
IMD	Indices of Multiple Deprivation
ITT	Intention To Treat
MCID	Minimum Clinically Important Difference
MDC	Minimum data collection
MRC	Medical Research Council
MRR	Medical Record Review
NHS	National Health Service
NICE	National Institute for Health and Care Excellence
PAG	Patient Advisory Group
PI	Principal Investigator
PIL	Participant Information Leaflet
PPIQ	Patient-Professional Interaction Questionnaire
QoL	Quality of Life
RCT	Randomised Controlled Trial
REC	Research Ethics Committee
ROS	Royal Osteoporosis Society
SAE	Serious Adverse Event
SD	Standard Deviation
SIMS	Satisfaction with medicines information
SOP	Standard Operating Procedure
TM	Trial Manager
TMG	Trial Management Group
TSC	Trial Steering Committee
UK	United Kingdom
VoI	Value of Information

## Data Availability

No data are associated with this article. Keele Data Repository: SPIRIT checklist for ‘A person-centred consultation intervention to improve shared decision-making about, and uptake of, osteoporosis medicines (iFraP): a pragmatic, parallel-group, individual randomised controlled trial protocol’. https://doi.org/10.21252/1vjh-5e30. Modified questionnaires used in the iFraP trial. Keele Data Repository. CC BY 4.0 license. Accessible: https://doi.org/10.21252/8895-pb55.
