# Supplementary material for: A person-centred consultation intervention to improve shared decision-making about, and uptake of, osteoporosis medicines (iFraP): a pragmatic, parallel-group, individual randomised controlled trial protocol
Source: NIHR Open Res. 2025 Oct 17;4:14. Originally published 2024 Apr 2. [Version 3] doi: 10.3310/nihropenres.13571.3 (PMC11320037; doi:10.3310/nihropenres.13571.3)
Supplement: Peer review history

# Reviewer report: Reviewer response for version 3

**DOI:** 10.3310/nihropenres.15382.r37875

**Published:** 2025-11-06

**Authors:** Alexander J. Rodríguez

**Affiliations:** 1 Edith Cowan University, Joondalup, Australia

Nil further input from me.

Is the study design appropriate for the research question?

Yes

Is the rationale for, and objectives of, the study clearly described?

Yes

Are sufficient details of the methods provided to allow replication by others?

Yes

Are the datasets clearly presented in a useable and accessible format?

Not applicable

Reviewer Expertise:

NA

I confirm that I have read this submission and believe that I have an appropriate level of expertise to confirm that it is of an acceptable scientific standard.

# Reviewer report: Reviewer response for version 2

**DOI:** 10.3310/nihropenres.14937.r33896

**Published:** 2025-01-28

**Authors:** Samuel Hawley

**Affiliations:** 1 University of Bristol, Bristol, England, UK

Thank you for the opportunity to review this very well-written study protocol for an RCT looking at the effectiveness of a combined intervention of an electronic decision support tool alongside clinician training in shared decision-making skills. Study outcomes include the ease of patient decision making with regards to osteoporosis medication initiation and short-term therapy persistence.

 There is strong rationale provided for the study - i.e. a context of suboptimal use of anti-osteoporosis medications, and the role of the clinician in closing the osteoporosis care gap.

 The study is well designed with details on all important aspects well justified. A detailed statistical analysis plan is yet to be finalized, but the details provided are sufficient and appropriate. I have the following minor points that the study’s investigators may have already considered, but might benefit from elaboration either in the current report or in future conduct of the study:

  

1. I note patients unable to comply with study procedures will be excluded. I wondered whether there were any particular study procedures envisaged that might preclude people from taking part? I suspect the study authors have already considered alternative procedures that might mitigate against too many people being excluded on this basis, but it could be worth briefly expanding on this given that the intervention is partly looking to improve decision making for those who might otherwise lack understanding/capacity to make informed decisions?
2. There is mention of site eligibility including the criteria of a minimum of 2 clinicians (presumably to allow each arm of the trial to be run). If I’ve understood correctly, is there also a need for a third clinician to act as a champion for the trial? If so, would it be true to say there will need to be a minimum of 3 clinicians per site?
3. If feasible, would blinding of study staff conducting assignment of clinician status to ‘intervention’ or ‘standard care’ help safeguard against clinician’s pre-existing preferences contributing to any contamination?
4. There is mention of a dental card at a couple of points, I might have missed the explanation for this but is that for all participants or just the intervention arm? Is it related to side-effects related to the jaw? Related to this, I noted a secondary outcome includes asking about concerns over common side-effects – would there be any rationale/scope to include fear of serious side effects alongside this? There is obviously good reason to expect the intervention to improve effectiveness of initiation, but it might be helpful to get insights into patient’s worries about serious side effects if these will potentially be discussed in greater detail during the baseline visit?
5. Very minor point – but typo/repeated words 'rather than' on page 11 toward end of bullet point starting “satisfaction with the consultation and…”

Is the study design appropriate for the research question?

Yes

Is the rationale for, and objectives of, the study clearly described?

Yes

Are sufficient details of the methods provided to allow replication by others?

Yes

Are the datasets clearly presented in a useable and accessible format?

Yes

Reviewer Expertise:

I am a statistician/epidemiologist with a background using routinely collected medical data for musculoskeletal epidemiology research.

I confirm that I have read this submission and believe that I have an appropriate level of expertise to confirm that it is of an acceptable scientific standard.

# Response

**Published:** 2025-10-09

**Authors:** Laurna Bullock

**Affiliations:** School of Medicine, Keele University, Stoke on Trent, England, UK

Thank you to the reviewer for the positive feedback. We have responded to each comment below, noting where minor changes to the manuscript have been made to address uncertainities raised.

**Reviewer comment 1:** I note patients unable to comply with study procedures will be excluded. I wondered whether there were any particular study procedures envisaged that might preclude people from taking part? I suspect the study authors have already considered alternative procedures that might mitigate against too many people being excluded on this basis, but it could be worth briefly expanding on this given that the intervention is partly looking to improve decision making for those who might otherwise lack understanding/capacity to make informed decisions?

**Author response: **Thank you for this comment. We designed the trial to reduce barriers to participation (e.g. by including a translated sentence in trial invite letters, acquiring costs for translation services, having paper documents for patient completion, in addition to electronic). One example where a patient would be ineligible is if they were unable to attend their face-to-face or telephone FLS appointment where the intervention is delivered.

**Reviewer comment 2:** There is mention of site eligibility including the criteria of a minimum of 2 clinicians (presumably to allow each arm of the trial to be run). If I’ve understood correctly, is there also a need for a third clinician to act as a champion for the trial? If so, would it be true to say there will need to be a minimum of 3 clinicians per site?

**Author response:** A minimum of two clinicians per site allows one clinician to be allocated to each trial arm. If one site only had two clinicians, the clinical champion role would be allocated to the clinician delivering the intervention to avoid contamination.

**Reviewer comment 3:** If feasible, would blinding of study staff conducting assignment of clinician status to ‘intervention’ or ‘standard care’ help safeguard against clinician’s pre-existing preferences contributing to any contamination?

**Author response: **Thank you for raising this important point. Many of the clinicians in the trial were not known to the study team at the time of allocation, thereby minimising any known ‘preferences’. Unblinded allocation to each trial arm allowed clinician characteristics to balanced e.g. considering seniority and years of experience.

**Reviewer comment 4a:** There is mention of a dental card at a couple of points, I might have missed the explanation for this but is that for all participants or just the intervention arm? Is it related to side-effects related to the jaw?

**Author response: **The dentist card is one of the intervention’s information resources and therefore will only be provided to patients in the intervention arm (as listed in the ‘iFraP intervention (iFraP-i)’ section). The dentist card aims to support ongoing information about osteoporosis medicines and dental health outside of FLS. The card includes personalised information about the patient’s medicine start date, expected treatment duration, and who the dentist should contact if they have questions. We have added more detail about the dentist card when describing the intervention components.

**Reviewer comment 4b:** I noted a secondary outcome includes asking about concerns over common side-effects – would there be any rationale/scope to include fear of serious side effects alongside this? There is obviously good reason to expect the intervention to improve effectiveness of initiation, but it might be helpful to get insights into patient’s worries about serious side effects if these will potentially be discussed in greater detail during the baseline visit?

**Author response: **At 2 week follow up, patients self-report specific osteoporosis values, including 5 bespoke questions about the relative perceived importance of osteoporosis medicine benefits and possible side effects and adverse events. One of the five bespoke questions asks patient participants: “How likely is it, that you would be put off taking this treatment, because of concerns about rare long-term issues with medicines such as jawbone problems”. Participants respond to this question using 5-response categories from “not at all” to “extremely”. We have made minor edits to the manuscript to make clear that the bespoke items listed are examples only.

**Reviewer comment 5:** Very minor point – but typo/repeated words 'rather than' on page 11 toward end of bullet point starting “satisfaction with the consultation and…”

**Author response: **Thank you for bringing this to our attention, we have rectified this mistake.

# Reviewer report: Reviewer response for version 2

**DOI:** 10.3310/nihropenres.14937.r34000

**Published:** 2025-01-10

**Authors:** Alexander J. Rodríguez

**Affiliations:** 1 Edith Cowan University, Joondalup, Australia

This protocol describes methods for a trial to examine if a decision support tool and training package provides utility in improving osteoporosis medicines uptake. This is an area of great need in the osteoporosis field. The methods and analysis are overall well described. Only two areas of concern should be addressed. First, how will the investigators overcome systematic bias in terms of patient recruitment given they are drawing on a pool of older adults referred owing to a fragility fracture. Randomisation at the patient level will just overcome imbalance between the two groups. Therefore, how will the investigators ensure their recruitment is generalisable to the osteoporosis/fragility fracture population. What efforts are in place to ensure representation of groups most at need and perhaps would be most hesitant to commence medications? Second, scant details are provided on the actual tool that will be used by the patients and clinicians together. Is this tool potentially modifiable or is it a proprietary product that was leveraged for the project?

Is the study design appropriate for the research question?

Yes

Is the rationale for, and objectives of, the study clearly described?

Yes

Are sufficient details of the methods provided to allow replication by others?

Yes

Are the datasets clearly presented in a useable and accessible format?

Not applicable

Reviewer Expertise:

Osteoporosis Vascular calcification Bisphosphonates

I confirm that I have read this submission and believe that I have an appropriate level of expertise to confirm that it is of an acceptable scientific standard, however I have significant reservations, as outlined above.

# Response

**Published:** 2025-10-09

**Authors:** Laurna Bullock

**Affiliations:** School of Medicine, Keele University, Stoke on Trent, England, UK

Thank you to the reviewer for their positive feedback. We have responded to each comment below in turn:

**Reviewer comment 1:** How will the investigators overcome systematic bias in terms of patient recruitment given they are drawing on a pool of older adults referred owing to a fragility fracture. Randomisation at the patient level will just overcome imbalance between the two groups. Therefore, how will the investigators ensure their recruitment is generalisable to the osteoporosis/fragility fracture population. What efforts are in place to ensure representation of groups most at need and perhaps would be most hesitant to commence medications?

**Author response: **In the UK, as part of Fracture Liaison Service normal NHS care, adults aged ≥50 years with a recent fragility fracture are systematically identified. This means that all identified patients that require a face-to-face or remote consultation in FLS where medicine may be discussed are mailed an initial invitation pack. However, it is possible that responders to the invitation may differ to non-responders. In efforts to minimise this, data collection materials and methods were carefully considered, with public contributors, in efforts to recruit a diverse trial sample. The public contributors recommended that the trial invite letter included a translated sentence in the five most commonly used languages and additional costs acquired for translation services. We also include paper completion of trial documents (in addition to electronic) to overcome barriers to participation.

**Reviewer comment 2:** Scant details are provided on the actual tool that will be used by the patients and clinicians together. Is this tool potentially modifiable or is it a proprietary product that was leveraged for the project?

**Author response: **Thank you for this comment. In the article’s introduction, we describe how the study team developed the tool (and other intervention components) guided by the Medical Research Council complex intervention development and evaluation framework. We also signpost the reader to a separate protocol outlining the in-depth intervention development and feasibility testing process. The results of each development study are also published and referenced in this article. The tool will be modified in response to the trial and process evaluation results.

# Reviewer report: Reviewer response for version 1

**DOI:** 10.3310/nihropenres.14731.r31549

**Published:** 2024-05-24

**Authors:** Li Shean Toh

**Affiliations:** 1 University of Nottingham, Nottingham, England, UK

The iFraP trial aims to improve shared decision-making (SDM) in the management of osteoporosis by integrating a computerized Decision Support Tool (DST), a clinician training package, and comprehensive information resources within UK Fracture Liaison Service consultations. This review examines the trial's background, objectives, methodology, and anticipated outcomes. The trial is novel in the aspect of patient centre aims which addresses a significant aspect of osteoporosis care: ensuring that treatment decisions align with patients' lives, beliefs, and values. By facilitating SDM, the iFraP intervention aims to enhance patient satisfaction and adherence to osteoporosis medication regimens. The iFraP trial is a pragmatic, parallel-group, individual randomized controlled trial with a nested mixed-methods process evaluation and health economic analysis. Some of the strengths are it uses a comprehensive approach by combining technological support with clinician education and patient information. It also has robust evaluation in place which employs a mixed-methods approach to capture a wide range of data, ensuring a thorough evaluation of the intervention. It also has a clear focus on SDM which directly addresses a critical aspect of patient-centered care. Some areas of suggestions is that the trial is conducted within the UK NHS context, which could have provided more details if the process evaluation would address more practical issues such as workload, funding, documentation time, logistics etc noting the pressures of the NHS. The other query is the follow-up duration, the follow-up period may be insufficient to fully capture long-term adherence and outcomes. It was also unclear in the recruitment whether there was consideration to ensure that deprived areas would be sought more to ensure there is a holistic view of the implementation of the services or other cultural competencies aspects. Overall, the iFraP trial is poised to provide valuable insights into the efficacy of integrating a DST with clinician training to enhance SDM in osteoporosis care. If successful, this intervention could be a significant step forward in personalizing osteoporosis treatment, improving patient outcomes, and optimizing healthcare resource utilization.

Is the study design appropriate for the research question?

Yes

Is the rationale for, and objectives of, the study clearly described?

Yes

Are sufficient details of the methods provided to allow replication by others?

Yes

Are the datasets clearly presented in a useable and accessible format?

Yes

Reviewer Expertise:

pharmacy practice and policy, osteoporosis, medicines management, intervention, mixed methods, health literacy

I confirm that I have read this submission and believe that I have an appropriate level of expertise to confirm that it is of an acceptable scientific standard.

# Response

**Published:** 2024-09-27

**Authors:** Laurna Bullock

**Affiliations:** School of Medicine, Keele University, Stoke on Trent, England, UK

We would like to express our thanks to the reviewer for reflecting on the strengths of iFraP and its opportunity to improve patient care. Below we have included each of the reviewer’s comments in turn, followed by our responses. Where needed, small revisions to the text of the article have been made. 

**Reviewer comment:** The trial is conducted within the UK NHS context, which could have provided more details if the process evaluation would address more practical issues such as workload, funding, documentation time, logistics etc noting the pressures of the NHS.

**Response:** The Medical Research Council’s guidance highlights the importance of considering how an intervention interacts with the context in which it is implemented. In line with this, the iFraP mixed methods process evaluation will explore how contextual factors affect the quantity and quality of intervention implementation (fidelity, dose and adaptation) and hypothesised intervention outcomes. The reviewer rightly acknowledges that relevant contextual factors may include practical issues, such as workload and resources. Further details about the mixed methods process evaluation will be published in a separate protocol.

**Reviewer comment**: The other query is the follow-up duration, the follow-up period may be insufficient to fully capture long-term adherence and outcomes.

**Response:** The trial’s final data collection occurs 3 months following the patient’s Fracture Liaison Service appointment. We anticipate that the 3-month period will provide insight into the initiation of osteoporosis drug treatments and short-term persistence and discontinuation of oral osteoporosis drug treatments (taken weekly). These findings will build on the limited evidence base to understand the clinical effectiveness of a shared decision-making intervention on uptake of preventative medicines.

 We do acknowledge, however, that the 3-month follow-up period may limit our understanding about long-term persistence or discontinuation, particularly for non-oral drug treatment options (which are administered less frequently e.g. monthly, 6 monthly or yearly).

 To acknowledge this, we have added the following sentence into the manuscript discussion: *“We do acknowledge, however, that the 3-month follow-up period of this trial may limit our understanding about long-term persistence or discontinuation of osteoporosis medicines.”*

**Reviewer comment:** It was also unclear in the recruitment whether there was consideration to ensure that deprived areas would be sought more to ensure there is a holistic view of the implementation of the services or other cultural competencies aspects.

**Response:** Thank you for this helpful comment. As part of the iFraP intervention development work, we conducted a national survey of Fracture Liaison Services (FLSs) ^1^. The survey findings captured service characteristics about potential trial sites. When selecting sites for the trial, the study team considered variation in FLS provision and the characteristics of the local population (e.g. socioeconomic status). Further information has been added to the protocol to acknowledge this: *“The selection of potential FLS sites will consider capturing variation in the FLS’s service provision (informed by the iFraP national survey of FLSs ^22^) and characteristics of their local population,”*

 Recruitment materials and methods were carefully considered, with public contributors, in efforts to recruit a diverse trial sample. The public contributors recommended that the trial invite letter included a translated sentence in the five most commonly used languages of each site and additional costs acquired for translation services. In a recent survey of patients attending rheumatology services (mean age 64.5), 20% of respondents reported not having access to an internet-enabled device and 18% reported limited health literacy ^2^. This demonstrated the need for paper surveys (in addition to online surveys) to overcome barriers to participation, despite the additional work required to operate hybrid recruitment and data collection methods. This is detailed in the protocol discussion.

 At a mid-point in patient recruitment, the Data Monitoring Committee (DMC) examined the characteristics of the patients recruited to the trial to determine if the sample is representative of the general population. In response to this interim review, an additional FLS site joined to trial (chosen because it served a larger proportion of underserved groups). This is detailed in Table 2, which provides an overview of protocol amendments since trial registration. This will be explored further as part of the trial results publications.

1. Bullock L, Abdelmagid S, Fleming J, et al. Variation in UK fracture liaison service consultation conduct and content before and during the COVID pandemic: results from the iFraP-D UK survey. *Arch Osteoporos* 2023; 19: 5.
2. Hider S, Muller S, Gray L, et al. Digital exclusion as a potential cause of inequalities in access to care: a survey in people with inflammatory rheumatic diseases. *Rheumatol Adv Pract*; 7. Epub ahead of print 30 December 2022. DOI: 10.1093/rap/rkac109

# Reviewer report: Reviewer response for version 1

**DOI:** 10.3310/nihropenres.14731.r31771

**Published:** 2024-05-18

**Authors:** Kathlene Camp

**Affiliations:** 1 University of North Texas Health Science Center, Fort Worth, USA

This is a well written research study protocol which has taken extended measures to design and describe steps to implement a RCT. 

 An initial question for consideration on the design is the 3-month assessment of adherence - will this be sufficient enough to assess adherence with all OP medications?  Will a 3-month time period be sufficient to assess discontinuation rates for non-oral OP medications or is this study suggesting only oral bisphosphonates are recommended for intervention. 

 Further details may be warranted to describe the qualitative component.  and not sure the health status- Euro-QoL EQ-5D-5L is an appropriate measure for this intervention, especially with the frequent delivery over a short period to time.

Is the study design appropriate for the research question?

Yes

Is the rationale for, and objectives of, the study clearly described?

Yes

Are sufficient details of the methods provided to allow replication by others?

Yes

Are the datasets clearly presented in a useable and accessible format?

Not applicable

Reviewer Expertise:

Primary understanding for background and clinical research design.

I confirm that I have read this submission and believe that I have an appropriate level of expertise to confirm that it is of an acceptable scientific standard.

## References

[1] : A person-centred consultation intervention to improve shared decision-making about, and uptake of, osteoporosis medicines (iFraP): a pragmatic, parallel-group, individual randomised controlled trial protocol. *NIHR Open Research* .2024;4: 10.3310/nihropenres.13571.1 10.3310/nihropenres.13571.1 39145101 PMC11320037

# Response

**Published:** 2024-09-27

**Authors:** Laurna Bullock

**Affiliations:** School of Medicine, Keele University, Stoke on Trent, England, UK

We would like to express our thanks to the reviewer for their time to provide insightful comment on this article. Below we have included each of the reviewer’s comments in turn, followed by our responses. Where needed, small revisions to the text of the article have been made, as described below. 

**Reviewer comment:** An initial question for consideration on the design is the 3-month assessment of adherence - will this be sufficient enough to assess adherence with all OP medications?  Will a 3-month time period be sufficient to assess discontinuation rates for non-oral OP medications or is this study suggesting only oral bisphosphonates are recommended for intervention. 

**Response:** The iFraP decision support tool, used by clinicians in Fracture Liaison Services, provides evidence-based treatment recommendations in line with clinical guidelines, including oral and non-oral drug treatment options. The clinician ultimately chooses which osteoporosis drug treatment(s) to discuss with the patient, based on their clinical judgement.

 The reviewer rightly highlights that the trial’s follow-up period is 3 months following the patient’s Fracture Liaison Service appointment. We anticipate that 3 months will be adequate to examine initiation as well as short-term persistence and discontinuation of oral osteoporosis drug treatments (taken weekly). We acknowledge that, because of the dosing regimens of non-oral drug treatment options (the time between doses are monthly, 6 monthly or yearly), the three month follow up period may not be sufficient to capture persistence and discontinuation of these drugs. Although this is a limitation, most patients who are recommended drug treatment in Fracture Liaison Services are recommended first-line oral drug treatments, administered weekly ^1^.

 To acknowledge this, we have added the following sentence into the manuscript discussion: *“We do acknowledge, however, that the 3-month follow-up period of this trial may limit our understanding about long-term persistence or discontinuation of osteoporosis medicines.”*

**Reviewer comment:** Further details may be warranted to describe the qualitative component. 

**Response:** Thank you for this comment. We appreciate that the information provided about the qualitative components of the mixed methods process evaluation are limited. A separate protocol will soon be published providing further details about the process evaluation.

**Reviewer comment:** Not sure the health status- Euro-QoL EQ-5D-5L is an appropriate measure for this intervention, especially with the frequent delivery over a short period to time. 

**Response: **The EQ-5D-5L will capture trial participant’s self-reported Health related Quality of Life at baseline (before attending their Fracture Liaison Service appointment) and 2-weeks and 3 months after their Fracture Liaison Service appointment. Self-reported health related quality of life is an essential element of the nested within-trial cost-effectiveness study. A separate protocol details this health economic study ^2^.

1. NICE (2019). Bisphosphonates for treating osteoporosis. https://www.nice.org.uk/guidance/ta464/resources/bisphosphonates-for-treating-osteoporosis-pdf-82604905556677
2. Siciliano M, Bathers S, Bentley I *et al.* Protocol for a trial-based economic evaluation analysis of a complex digital health intervention including a computerised decision support tool: the iFraP intervention [version 1; peer review: awaiting peer review]. *NIHR Open Res* 2024, **4**:15 ( https://doi.org/10.3310/nihropenres.13575.1)
